# Anemia among Patients Attending Anti-retroviral Therapy at a Tertiary Care Center: A Descriptive Cross-sectional Study

**DOI:** 10.31729/jnma.6318

**Published:** 2021-12-31

**Authors:** Bibhant Shah, Lochan Karki, Rajesh Kumar Mandal

**Affiliations:** 1 Department of Internal Medicine, National Academy of Medical Sciences, Bir Hospital, Kathmandu, Nepal; 2 Department of Internal Medicine, Bheri Hospital, Nepalgunj, Nepal

**Keywords:** *anemia*, *anti-retroviral therapy*, *human immunodeficiency virus*, *prevalence*

## Abstract

**Introduction:**

Hematologic abnormalities are among the most common complications of infection with Human Immunodeficiency Virus. These abnormalities are due to: impaired hematopoiesis, immune mediated cytopenias and altered coagulation mechanisms. Anemia is the most frequent, however, leukopenia, lymphopenia, and thrombocytopenia have also been observed. The aim of the study was to find the prevalence of anemia in patients attending anti-retroviral therapy at a tertiary care center of Nepal

**Methods:**

The study was a descriptive cross-sectional study conducted from August 2018 to August 2019 in patients attending anti-retroviral therapy at a tertiary care hospital. Ethical approval was obtained from the Institutional Review Board of National Academy of Medical Sciences before starting the study (Reference number 267). Convenient sampling was used for this study. Data were analysed using the Statistical package for Social Sciences version 20. Point estimate at 90% confidence interval was calculated along with frequency and proportion for the binary data.

**Results:**

The prevalence of anemia among patients attending anti-retroviral therapy centers in our study was found in 29 (58%) (46.55-69.45 at 90% Confidence Interval). Out of those patients, 20 (63%) were male and 9 (50%) were female. The mean hemoglobin value was 11.946±2.51g/dl.

**Conclusions:**

The prevalence of anemia among patients attending antiretroviral therapy in our study was found to be high which is consistent with the findings of other similar international studies. These patients should be routinely monitored and treated for the occurrence of hematological abnormalities.

## INTRODUCTION

Hematological abnormalities are among the most common clinicopathological manifestations of Human Immunodeficiency Virus (HIV) infection. It is associated with impaired hematopoiesis, immune mediated cytopenias and coagulopathies, particularly in the later part of the disease.^1^ The most prevalent hematological disorder observed with HIV infection is anemia and it is particularly high in patients with late stages of disease and a reduced CD4 cell count.^2-4^

Such hematological problem hinder the treatment of both, primary viral infection and the opportunistic infections.^5^ They are associated with increased morbidity and mortality, affecting their quality of life.^6^ Tests like CD4 count is not readily available in our country. Complete blood test are available even at local levels. It can be a great tool for monitoring anemia in HIV patients.

This study aims to find the prevalence of anemia in patients attending anti-retroviral therapy at a tertiary care center of Nepal.

## METHODS

This was a descriptive cross-sectional study conducted in patients attending anti-retroviral therapy at antiretroviral therapy center at National Academy of Medical Sciences (NAMS), Bir hospital, Kathmandu from August 2018 to August 2019. Ethical approval was obtained from the Institutional Review Board (IRB) of National Academy of Medical Sciences (NAMS) before starting the study (Ref. No. 267). Inclusion criteria were age group between 20 and 55 years, patients with HIV infection proven by ELISA, patients on ART, patients providing dated written informed consent, patient's registered to ART center and those with Nepalese citizenship. Exclusion criteria include patient with age group between 20 and 55 years, patients on ART with acute systemic illness, patients already having disease causing anemia co-occurring with HIV. e.g. rheumatoid arthritis, chronic infection like tuberculosis, alcoholics (as defined by DSM - IV criteria for alcohol dependence), chronic kidney disease (eGFR<30ml/min for duration of 3 months), drugs intake (phenytoin, methotrexate), hematological disease. The convenient sampling method was used for data collection, and the sample size was calculated as follows:

n = Z^2^ × p × q / e^2^

  = (1.64)^2^ × 0.77 (1-0.77) / 0.10^2^

  = 48

Where,

n= minimum required sample size
Z= 1.64 at 90% Confidence Interval (CI)
p= past prevalence prevalence of anemia in HIV patients taken from a previous study,77%^8^
q= 1-p
e= margin of error, 10%

Hence, the sample size calculated for this study is 48 patients. However, 50 cases were enrolled in this study.

A written informed consent was obtained from all the participants. The demographic information was recorded. The CD4 was measured by flow cytometry.

Statistical package for social sciences (SPSS) version 20 was used for data collection and analysis. Descriptive statistics like frequency, percentages were calculated.

## RESULTS

The prevalence of anemia among patients attending anti-retroviral therapy centers in our study was found to be 29 (58%) (46.55-69.45 at 90% Confidence Interval). The mean hemoglobin value among these patients was 11.946±2.51g/dl. Out of those patients, 20 (68.97%) were male and 9 (31.03%) were female (Table 1).

**Table 1:** Gender wise distribution.

Gender	n (%)
Male	34 (53.30)
Female	30 (46.80)
**Total**	**29 (100)**

A total of 50 HIV infected patients were enrolled in the study consisting 32 (64%) male and 18 (36%) female. The mean age was found to be 37.44±9.058. (Table 2).

**Table 2:** Age Distribution of astudy participants anemia among patients attending anti-retroviral therapy (n=29).

Age in years	Male n (%)	Female n (%)	Total n (%)
21-30	6 (0.26)	3 (0.10)	9 (31.03)
31-40	8 (0.28)	4 (0.14)	12 (41.37)
41-50	4 (0.14)	2 (0.7)	6 (20.69)
51-55	2 (0.7)	0	2 (6.89)
**Total**	**20 (69)**	**9 (31)**	**29 (100)**

## DISCUSSION

A total of 50 patients were enrolled out of which 32 were male and 18 were female. Anemia (defined as hemoglobin <12gm/dl and <13gm/dl for female and male respectively) was found in 58% patients out of which 63% were male and 50% were female. Our results on prevalence of anemia showed comparable results with other studies from India 46%, 74%, 65.5% respectively.^9-11^ This is also comparable to the 20 to 60% in studies done in America and Europe.^12^ The occurrence of anemia was found to be more common among male. This male predominance was supported by another study where prevalence of anemia was 33.1% in woman and 40.5% in man.^13^ Anemia was reported as a consistent hematological abnormality in HIV/AIDS by Ogun, et al.^14^ Mitsuyasu, et al. and Zon, et al. detected anemia in approximately 10 to 20% at initial presentation and in 70 to 80% over the course of HIV infection.^15-16^ These studies show higher prevalence than that of our study.

The prevalence of anemia was found to be lower 37.7% in Brazilian study which contradict to our study.^17^ Possible explanations for this finding could be the good adherence of patients to medical follow-up and treatment (more than 80% of patients regularly using HAART drugs), and improvements in social and nutritional conditions that have occurred in Brazil, over recent decades.^17^ Anemia is an independent predictor of survival in HIV infection and mortality is increased by 60% in anemia patients with CD4 <200/microlitre.^16^ There exists a negative association between the prevalence of anemia and CD4 counts, indicating that the proportion of patients with anemia increased as severity of HIV increased, suggesting decreasing immune function have direct impact on Hemoglobin synthesis.^18^ Degree of anemia can be easily assessed in any rural clinical setting.^18^ In the present study 58% had anemia, out of which 70% had CD4 <200/microlitre. This highlights the possibility of increased mortality in our set of patients. The institution of HAART in these patients has the chance of converting them into transfusion dependent anemia patients.

The common cause of anemia in HIV infected patients is bone marrow failure and use of zidovudine. Other causes like autoimmune antibodies to hemopoietic precursors, opportunistic infections like Cytomegalovirus (CMV), B19 parvovirus, or Mycobacterium avium-intracellulare suppressing erythropoiesis, hemolytic anemia, anemia related to gastrointestinal bleeding, decreased erythropoietin levels, nutritional anemia due to vitamin B12 and iron deficiency resulting from malabsorption or malnutrition have also been stated.^18^

The limitation of our study was that it is a hospital based study with small sample size, no randomization, short time duration, and various associated comorbidity conditions not taken into account during the study. Similarly, this study cannot be generalized to whole Nepal as it is conducted in a single hospital of Nepal.

## CONCLUSIONS

The prevalence of anemia among patients attending anti-retroviral therapy in our study was found to be high which is consistent with the findings of other similar international studies. The basic hematological parameters like haemoglobin can be used as a prospective screening test to assess the severity and progression of HIV infection when CD4 count is not available, especially in developing countries like Nepal where financial resources are slightly limited.
